# Genetic evidence of multiple invasions and a small number of founders of Asian Palmyra palm (*Borassus flabellifer*) in Thailand

**DOI:** 10.1186/s12863-017-0554-y

**Published:** 2017-10-12

**Authors:** Kwanjai Pipatchartlearnwong, Akarapong Swatdipong, Supachai Vuttipongchaikij, Somsak Apisitwanich

**Affiliations:** 1 Department of Genetics, Faculty of Science, Kasetsart University, 50 Ngam Wong Wan Road, Chatuchak, Bangkok, 10900 Thailand; 2 Center of Advanced studies for Tropical Natural Resources, Kasetsart University, 50 Ngam Wong Wan Road, Chatuchak, Bangkok, 10900 Thailand; 3 Special Research Unit in Microalgal Molecular Genetics and Functional Genomics (MMGFG), Department of Genetics, Faculty of Science, Kasetsart University, 50 Ngam Wong Wan Road, Chatuchak, Bangkok, 10900 Thailand; 4 School of Science, Mae Fah Luang University, Chiang-Rai, 57100 Thailand

**Keywords:** Arecaceae, Expressed sequence tag- simple sequence repeat (EST-SSR), Genetic diversity, Genomic-simple sequence repeat (genomic SSR), Microsatellite marker, Minimum founder

## Abstract

**Background:**

*Borassus flabellifer* or Asian Palmyra palm is an important crop for local economies in the South and Southeast Asia for its fruit and palm sugar production. Archeological and historical evidence indicated the presence of this species in Southeast Asia dating back at least 1500 years. *B. flabellifer* is believed to be originated in Africa, spread to South Asia and introduced into Southeast Asia through commercial routes and dissemination of cultures, however, the nature of its invasion and settlement in Thailand is unclear.

**Results:**

Here, we analyzed genetic data of 230 *B. flabellifer* accessions across Thailand using 17 EST-SSR and 12 gSSR polymorphic markers. Clustering analysis revealed that the population consisted of two genetic clusters (STRUCTURE K = 2). Cluster I is found mainly in southern Thailand, while Cluster II is found mainly in the northeastern. Those found in the central are of an extensive mix between the two. These two clusters are in moderate differentiation (*F*_ST_ = 0.066 and *N*_M_ = 3.532) and have low genetic diversity (H_O_ = 0.371 and 0.416; A_R_ = 2.99 and 3.19, for the cluster I and II respectively). The minimum numbers of founders for each genetic group varies from 3 to 4 individuals, based on simulation using different allele frequency assumptions. These numbers coincide with that *B. flabellifer* is dioecious, and a number of seeds had to be simultaneously introduced for obtaining both male and female founders.

**Conclusions:**

From these data and geographical and historical evidence, we hypothesize that there were at least two different invasive events of *B. flabellifer* in Thailand. *B. flabellifer* was likely brought through the Straits of Malacca to be propagated in the southern Thailand as one of the invasive events before spreading to the central Thailand. The second event likely occurred in Khmer Empire, currently Cambodia, before spreading to the northeastern Thailand.

**Electronic supplementary material:**

The online version of this article (10.1186/s12863-017-0554-y) contains supplementary material, which is available to authorized users.

## Background

Biological invasions occurred both naturally and unnaturally, and the rate of invasions has been elevated since the rise of global trades [1–3]. Although invasive species theoretically pose problems for the well-being of natural communities and ecosystems [4–7], a large number of invasive plants have been deliberately introduced to new areas due to various beneficial purposes including medicines, ornament, and food [8–10]. *Borassus flabellifer* (Asian Palmyra palm), as of African origin [11], is one of the invasive plants in Thailand and Southeast Asian countries. Although it is sometimes considered as indigenous to the Indian subcontinent, it is likely that *B. flabellifer* was spread out from Africa to South Asia before being introduced into Southeast Asia through commercial routes in the past [12, 13]. In Southeast Asian countries, *B. flabellifer* is one of the oldest domesticated fruit crops estimating that it has been in Thailand since 1500 years ago. Almost all parts of the plant are used by the locals, and its fruit is widely consumed. Especially, sap from the inflorescence flower is the source for palm sugar production and alcoholic beverages. As a result, *B. flabellifer* was distributed and grown in many regions of Thailand in various soil and climatic conditions.

*B. flabellifer*, a dioecious monocotyledonous woody perennial tree in the family Arecaceae, is a massive palm with a single stem reaching 30 m in height and large fan-shaped leaves spanning 4–6 m in diameters [11]. *B. flabellifer* grows very slow, taking up to 12–20 years to reach its maturity and produce its first inflorescence flowers. Sex determination based on plant morphology is not possible, and that based on molecular markers is currently unachievable. Previous genetic studies using various types of DNA markers showed that *B. flabellifer* populations have very low genetic diversity [14–17], and this was concerned as a potential threat to a sustainable use as the species is in decline through extensions of farmland, urbanization and its extremely long juvenile stage.

A standing question is whether the narrow genetic diversity of *B. flabellifer* should be a key consideration for its agricultural sustainability or it should be acknowledged that this had occurred through selection and domestication processes a long time ago. Here, we analyzed the genetic diversity and population structure of *B. flabellifer* across Thailand using microsatellite markers. The phylogeography was also observed, and the minimum numbers of founder individuals introduced into Thailand were estimated. Geographical and historical evidence was taken into account to form theoretical invasive events of *B. flabellifer* in Thailand and Southeast Asia.

## Methods

### Plant materials, gDNA isolation and SSR amplification

To obtain genetic data, *B. flabellifer* population was analyzed by 17 EST-SSR and 12 gSSR polymorphic markers (Table 1). These microsatellite markers, which were originally developed based on oil palm [17, 18], have been tested and evaluated for their transferability and polymorphism in *B. flabellifer* population. Young leaf samples were collected from 230 *B. flabellifer* accessions located throughout Thailand (31, 139 and 60 plants from southern, central and northeastern Thailand, respectively, see Additional file 1). An accession of oil palm was used as an outgroup. Total DNA was isolated from leaf samples using a modified CTAB extraction method [19]. PCR amplification was conducted as follows: an initial denaturation step of 5 min at 94^o^ C, followed by 35 cycles of three steps, 30 s at 94^o^ C, 1.30 min at the specific annealing temperature for each primer pair and 30 s at 72^o^ C, and a final extension step at 72^o^ C for 8 min. PCR products were resolved using 6% polyacrylamide gel electrophoresis and stained with silver nitrate (see Additional file 2 for representative polyacrylamide electrophoresis gels for the polymorphic loci). PCR fragments obtained for EST-SSRs and gSSRs were size-estimated based on the Low Molecular Weight DNA Ladder (Biolabs® Inc., New England).

**Table 1:** Summary of the polymorphic microsatellite loci used in the *B. flabellifer* population

Primer	Repeat motif	Primer sequence (5′-3′)	Tm (°C)	Allele size (bp)	PIC
ESSR75	(AAG)_5_	F:AGATGGTTGGAGATTTCATGGT R:AACTTGAGGGTGCCATTACAAG	60	270–285	0.38
				175–190	0.44
ESSR76	(AGC)_5_	F:CCATACCAGCAGAAGAGGATGT R:CTGAAGGTCATAGGGGTCTCTG	60	350–365	0.52
				190–205	0.56
ESSR82	(GCT)_5_	F:CCCTCGACACCCATAGTTATTT R:CTCGATTTCTGGCCTCTCATAC	60	200–215	0.39
ESSR332	(AT)_6_	F:AGTTAATGTGTCAGGGCCAGTT R:CTTGGTTCACTTGGGTGTGTC	60	230–242	0.42
ESSR553	(A)_19_	F:ATAAATTGTGCGAGGGGAAAAC R:AGATCCGCGACAGGTCTTAAC	60	220–239	0.37
				125–144	0.34
ESSR566	(AG)_7_	F:GTGTCATCAAATTCGGTCCTTT R:CGGTTCTTCTGCTGCTCTACTT	60	240–254	0.44
				125–139	0.56
ESSR609	(GA)_7_	F:AGGCGGTGATGAAGATGAAG R:CTCCTCTCAAACAGAGTGGGAT	59	150–164	0.4
ESSR650	(AG)_14_	F:GCCTTTTCTGGTTAATGGACTG R:GTTTGTCTATGGATGATTGTGAGG	59	200–228	0.32
ESSR652	(GAG)_6_	F:CATACCGTCACCACTCAGAAAC R:GCCGTCATTCTACCAGTTGAG	60	150–168	0.33
ESSR673	(GGC)_8_	F:TTCTGGCTACGAGCATAAGGA R:TCAATAACCCTGGCTAAACACA	59	150–168	0.23
				75–113	0.36
ESSR681	(AAAT)_5_	F:TCTGAATTGTCGGAGTGGC R:CATCCTTGCGTAAACAAAAGAG	59	350–370	0.49
				130–150	0.56
mEgCIR2332	(GA)_14_	F:GAAGAAGAGCAAAAGAGAAG R:GCTAGGTGAAAAATAAAGTT	55	250–278	0.38
mEgCIR3295	(GT)_7_ (GA)_23_	F:TGCCTCCAGACAATCAC R:GTAAGGCTTAACCAGATAAC	55	300–360	0.62
				250–310	0.16
mEgCIR3311	(GA)_15_	F:AATCCAAGTGGCCTACAG R:CATGGCTTTGCTCAGTCA	55	180–210	0.31
mEgCIR3413	(GA)_18_	F:AAAGCTATGGGGTGAAAGAT R:TGGATAAGGGCGAGAAGAGA	55	350–386	0.27
				250–286	0.44
mEgCIR3477	(GA)_22_	F:CCTTCAAGCAAAGATACC R:GGCACCAAACACAGTAA	55	250–294	0.15
mEgCIR3592	(GA)_20_	F:GAGCCAAAACAGACTTCAA R:ACCGTATATGACCCCTCTC	55	230–270 150–190	0.46 0.3
mEgCIR3755	(GA)_15_	F:GCTCACCAAAAAGTGTTAAGTC R:AGTTTCAACGGCAGGTATAT	55	340–370 140–170	0.02 0.1
mEgCIR3788	(GA)_18_	F:TTGTATGACCAAAGACAGC R:AGCGCAACATCAGACTA	55	180–216	0.43

### Genetic diversity and population structure analyses

All loci were tested for linkage disequilibrium (LD) using PowerMarker version 3.25 [20]. The sequential Bonferroni correction [21] was performed according to the multiple tests. The Hardy-Weinberg equilibrium (HWE) was conducted using POPGENE version 1.31 [22]. PowerMarker V3.25 was used to determine polymorphism information content (PIC) based on Botstein et al. (1980) [23].

EST-SSR and gSSR data were used to infer the most likely number of population genetic clusters (K), based on a Bayesian approach implemented in STRUCTURE version 2.3.4 [24]. Each of individual *B. flabellifer* was initially grouped according to the sampling locations: provinces and geographical parts of Thailand. Assuming a population admixture model, 100,000 burn-ins and 100,000 Markov chain Monte Carlo (MCMC) replicates for K varied from 1 to 10 were performed. Twenty independent runs were performed for each K. The best K was then inferred based on ln*P(D)* and delta K [25]. A hierarchical analysis of molecular variance (AMOVA) was performed using Arlequin version 3.1 [26] to test for significance of grouping based on provinces and regional parts of Thailand and that of genetic clustering using STRUCTURE. The level of polymorphism was determined by using all 29 polymorphic loci. The observed (*H*_*o*_) and expected (*H*_*E*_) heterozygosity, the number of observed alleles and the number of effective alleles were calculated using POPGEN version 1.31. Allelic richness (A_r_) and pairwise *F*_ST_ among groups were calculated using FSTAT version 2.9.3.2 [27]. The number of migrants (*N*_M_) was estimated using an equation according to Wright [28]: *N*_M_ = 1(1/*F*_ST_-1)/4.

### Minimum number of founders

Although a number of methods for estimating a minimum number of founders based on genetic data are available, these are unsuitable for our dataset as they require genetic information from the source population and assume no admixture between introduced populations. Thus, to estimate the minimum number of founders, we used an approach developed by Rasner et al. [29], which requires empirically information from observed microsatellite alleles in each cluster of the studied species. Information based on best grouping identified using AMOVA result (i.e. maximum *F*_CT_) was used.

Custom-written scripts (see Additional file 3) based on program R 3.3.0 for simulating different numbers of founder genotypes was run for 10,000 replicates to find the minimum number of founder individuals. This was performed in two criteria: simulating using the complete set of genotypes contain all the microsatellite alleles in the dataset and simulating using a data set excluding the low frequency alleles (<0.02). In accounting for the effects of sampling bias and genetic drift in small populations, the R scripts were run in three following setting: (1) randomly resampling alleles—independently for each locus—without replacement from the data set, (2) resampling from allele frequency distribution at each locus and (3) resampling from allele frequency distributions by assuming equal allele frequencies at each locus.

## Results

### EST-SSR and gSSR analyses of a *B. flabellifer* population in Thailand

In total, 17 EST-SSR and 12 gSSR polymorphic loci in 230 *B. flabellifer* individuals were analyzed. Sequential Bonferroni correction was applied due to the multiple tests, and the LD test revealed that these 29 loci were not physically linked (Table 1). Thus, all loci were used for downstream genetic analysis. The PIC value across the polymorphic loci was 0.37. The PIC values of 17 EST-SSRs were 0.23–0.56 (average of 0.42), and those of 12 gSSRs were 0.02–0.62 (average of 0.32).

### STRUCTURE clustering of *B. flabellifer*

**Fig. 1:**
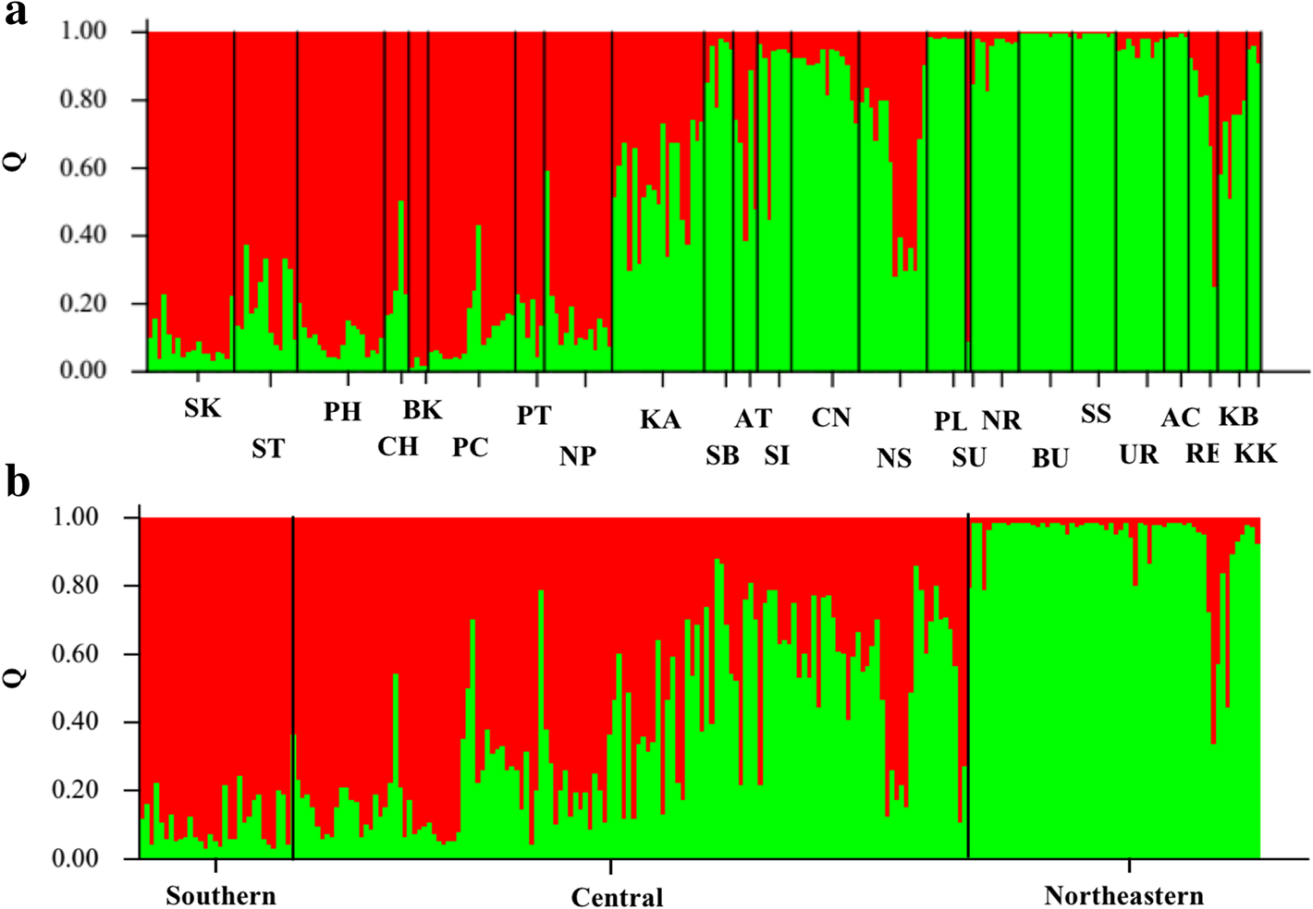
Genetic clustering of 230 *B. flabellifer* individuals based on STRUCTURE analyses using information of sampling sites by 24 provinces (**a**) and three regions (**b**). The best K (K = 2) is indicated for both clusters

The genotypic data of the *B. flabellifer* based on the 29 loci were subjected to clustering analysis using STRUCTURE program. Initially, the best K from the genotypic data following Pritchard et al. [24] was unable to be obtained, because the ln*P*(D) increased continually as the K increased (data not shown). The delta K statistics based on Evanno et al. [25] was then applied and indicated the best K = 2 for a STRUCTURE clustering analysis using sampling locations based on either provinces or geographical areas (Fig. 1).

**Fig. 2:**
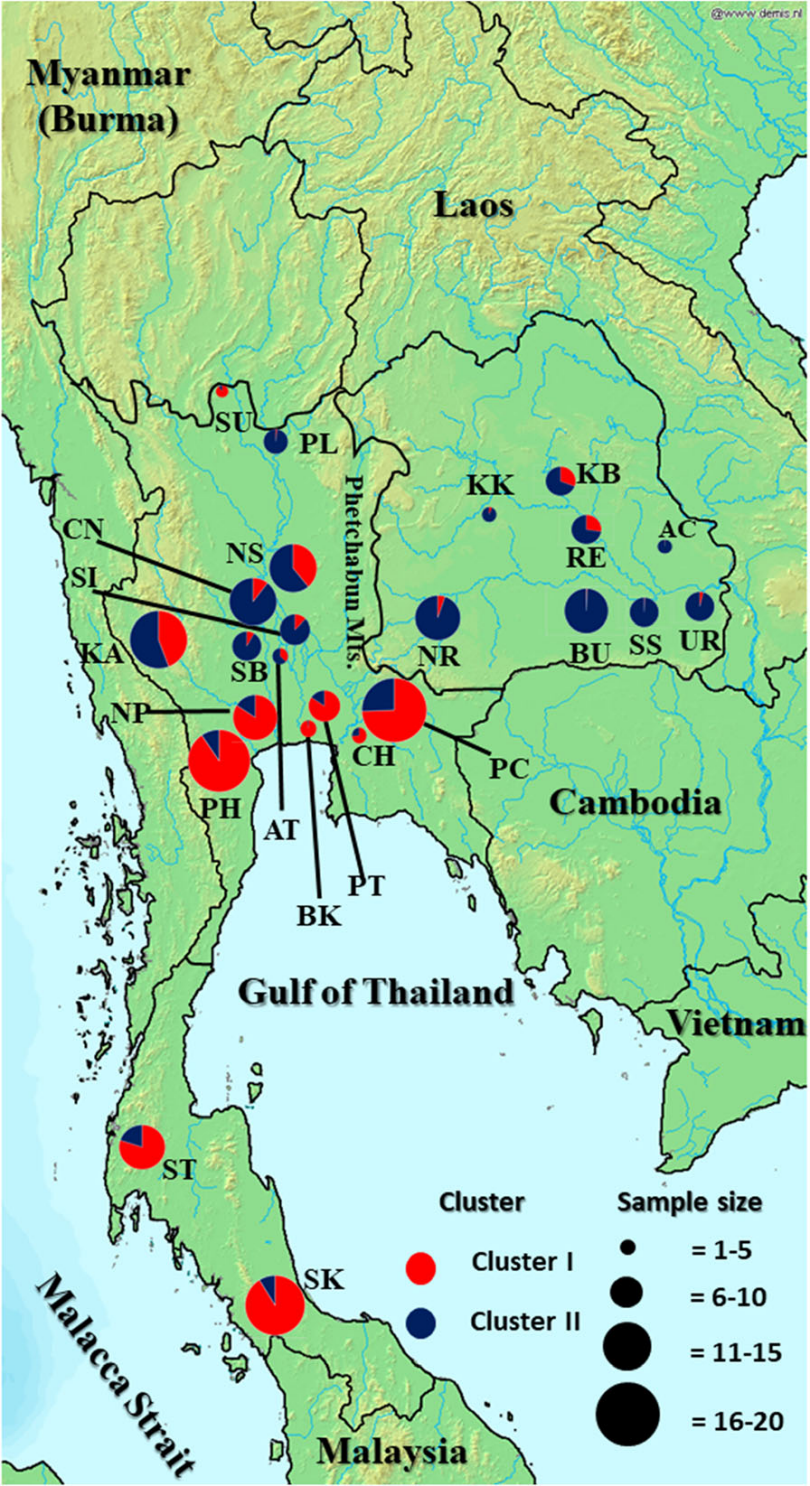
Geographical distribution of the STRUCTURE clusters (K = 2) *B. flabellifer* in Thailand. The map depicted here was taken from Wikimedia Commons. Colors in pie charts represented the different clusters. The sizes of pie chart represented sample sizes. For abbreviation of provinces see Additional file 1

Among the four alternative grouping types, the hierarchical analysis of molecular variance (AMOVA) showed that grouping based on STRUCTURE assisted by the three sampling regions was the most suitable for these samples as indicated by the highest *F*_CT_ (max *F*_CT_ = 0.06266; Table 2). It indicated that the genetic variation of the *B. flabellifer* in Thailand was highest within the populations (86.13%) followed by among populations (7.6%) and smallest among clusters (6.27%). Therefore, the grouping as two clusters was used for subsequent analyses. By mapping the clusters and sample sizes, it revealed that *B. flabellifer* individuals from cluster I dominate southern Thailand, while those from cluster II dominate the northeastern (Fig. 2). Individuals from the two clusters were found equally in the central region and mixed in a number of provinces. The ratio of the two clusters found within the same sampling sites was found varied greatly among provinces.

**Table 2:** Hierarchical analysis of molecular variance (AMOVA) of four grouping types based on sampling locations or STRUCTURE clustering assisted by sampling locations

Grouping	Source of variation	Total variation	Percentage	FCT
24 groups based on provinces	Among clusters	0.20839	4.13	0.04131 (*p*<0.0001)
	Among populations within clusters	0.40812	8.09	
	Within populations	4.42753	87.78	
	Total	5.04405		
3 groups based on geographical areas	Among clusters	0.21782	3.42	0.03416 (*p*<0.0001)
	Among populations within clusters	0.62788	9.85	
	Within populations	5.53157	86.74	
	Total	6.37727		
2 clusters based on STRUCTURE assisted by sampling provinces	Among clusters	0.26121	5.19	0.05192 (*p*<0.0001)
	Among populations within clusters	0.36636	7.28	
	Within populations	4.40375	87.53	
	Total	5.03132		
2 clusters based on STRUCTURE assisted by geographical areas	Among clusters	0.31992	6.27	0.06266 (*p*<0.0001)
	Among populations within clusters	0.38812	7.6	
	Within populations	4.39782	86.13	
	Total	5.10586

### Genetic diversity of the *B. flabellifer* based on STRUCTURE clustering

The microsatellite data of the two clusters based on STRUCTURE clustering were separately analyzed. It showed that 120 and 110 individuals of the cluster I and II, respectively, had low numbers of observed alleles_,_ effective alleles, observed heterozygosity and heterozygosity (Table 3). Estimated allelic richness based on a minimal sample size of 96 diploid individuals were 3.00 and 3.19 for the cluster I and II, respectively. This result indicated that both cluster I and II have very low genetic diversity. Each cluster was found to be highly significantly deviated from HWE (*p* < 0.0001). This is most likely because the *B. flabellifer* is dioecious, allowing gene flow between the two clusters within the same sampling sites. The *F*_ST_ and *N*_M_ values between both clusters were 0.066 and 3.532, respectively, indicating that these clusters are in moderate differentiation with a moderate gene flow.

**Table 3:** Genetic diversity across 29 polymorphic microsatellite loci on the two STRUCTURE clusters of the *B. flabellifer* population

Cluster	n	N_A_	N_E_	H_o_	H_e_	A_R_
1	120	3.0345	1.7494	0.3705	0.392	2.998793
2	110	3.2069	1.984	0.4163	0.4431	3.191897
Mean	115	3.1207	1.8667	0.3934	0.4176	3.0953

Sample number (n), the number of observed alleles (NA), the number of effective alleles (NE), the observed (Ho) and expected (HE) heterozygosity and allelic richness (AR) base on a minimal sample size of 96 diploid individuals are presented

### The minimum number of *B. flabellifer* founders in Thailand

As indicated by historical evidence and supported by the low genetic diversity, *B. flabellifer* was introduced into Thailand, and, thus, could be originated from a considerably small number of founders. The number of *B. flabellifer* founders was estimated using the microsatellite data. As expected, the total number of alleles included in the analysis affected the estimated minimum number of founders (Table 4). The minimum numbers were estimated based on the genotypes, which were simulated by resampling from the allele frequency distributions. When resampling all empirical alleles in the dataset, an inclusion of all alleles potentially resulted in an overestimation of the number of the original founders of the two clusters (34 and 25 for cluster I and II, respectively). After removing the low frequency alleles, 13 and 9 founders were estimated for the cluster I and II, respectively. Depending on the reference dataset used, estimated numbers for the cluster I varied between 13 and 34 founders, and the cluster II varied between 9 and 25 founders. Finally, the simulation assuming equal allele frequencies at all loci consistently gave the lowest number of founders; four founders for both two clusters when using the dataset with all alleles and three founders for both clusters when using the dataset without low frequency alleles.

**Table 4:** Estimated minimum numbers of founders required to introduce all empirically observed microsatellite alleles into each STRUCTURE cluster of the *B. flabellifer* population

Cluster	Resampling of alleles	Allele frequency	Equal frequency
(a)			
Cluster I	34	39	4
Cluster II	25	27	4
(b)			
Cluster I	13	10	3
Cluster II	9	9	3

The simulations were performed using (a) the complete set of genotypes assigned to each cluster or (b) excluding low frequency alleles (<0.02). Estimates were obtained using three different approaches: resampling of alleles (without replacement), empirical allele frequency, and equal allele frequency

## Discussion

Currently, there are two hypotheses regarding the origin of *B. flabellifer.* First*,* although there is so far no report on the presence of this species in Africa, it is thought to be originated in this continent [11] and then spread into India at least 2500 year ago, based on a report by the Greek historian Megasthenes, ambassador to the court of Chandragupta [12, 13]. An alternative hypothesis is that *B. flabellifer* is native to South Asia, Southeast Asia, New Guinea and Tropical Africa (Morton 1988). However, the recent evidence favors the first hypothesis as five of six *Borassus* species are found in Africa, and, specifically, *B. aethiopum* and *B. akeassii* have similar morphology to that of *B. flabellifer* [30–32]. It can be postulated that *B. flabellifer* was spread to India and its subcontinent and later to Southeast Asia because of its values in palm sugar production and alcohol products.

*B. flabellifer* was brought to Southeast Asia most likely through the commercial routes and dissemination of cultures a long time ago. Based on our results, we hypothesize that the *B. flabellifer* was introduced into the areas, which are currently parts of Thailand, from two directions. Based on geographical reasons and historical commercial routes, one could be initially introduced into the southern part of Thailand, possibly from the Straits of Malacca as the world important shipping route since the past [33]. Another was likely introduced into the northeastern, possibly through Vietnam, Cambodia or Laos. These neighboring countries had long been interconnected for transferring and sharing cultures and goods since the historical time. Furthermore, our result showing a wide separation between the two clusters reflects the fact that there are mountain ranges that obstructed the movement between two clusters in the northeastern and the rest of Thailand. Nevertheless, the mix between the two clusters might have occurred gradually at the later time.

Historical and archeological evidence indicated the presence of *B. flabellifer* in Thailand since at least 1500 years ago. This evidence includes the discovery of a stone sealing in the Dvaravati period (central Thailand ~1500 years ago) showing a man climbing a palm tree [34] and an identification of *B. flabellifer* pollen in archeological specimens aged ~1500 years in Songkhla province, southern Thailand [35]. We speculate that the southern Thailand was the introduction route of the cluster I via the Straits of Malacca, rather than Myanmar. This is because areas of the two countries are separated by high and long mountain ranges.

Our finding of the second settlement in the northeastern Thailand was firstly unexpected. Nonetheless, there is a number of evidence supporting this finding, including archeological studies in Angkor Borei in southern Cambodia that found pollen and tissues of *B. flabellifer* dating back approximately 1400–1500 years [36, 37]. Although there are also ancient scripts describing the presence of *B. flabellifer* in Laos in the past 1500 years [38, 39], we envisage that *B. flabellifer* in the northeastern Thailand was likely introduced from the areas, where it is now Cambodia, rather than Laos or Vietnam. This is because our result showed that the cluster II is concentrated at the lower part of the northeastern Thailand next to the Cambodia border, which has no major natural barriers. Noting that, in 7th centuries, this area in the northeastern Thailand belonged to the Khmer Empire. Furthermore, because there are long mountain ranges that separate between Vietnam and Laos, it is unlikely that *B. flabellifer* was introduced into Thailand through Vietnam, Laos and Mekong river. More samples from the neighboring countries are required to obtain a clearer picture of the *B. flabellifer* introduction route in Southeast Asia.

Since *B. flabellifer* requires 12–20 years to reach its flowering stage and considering the 1500 years of settlement, this means that the species has been reproducing in Thailand for at least 125 generations. Thus, it is not surprising that our observed allele diversity was very low when considering the number of generation and genetic variation indexes. Likewise, no clear phenotypic variations among the population have been identified. Furthermore, the genetic study of this species in India, as thought to be the origin of *B. flabellifer* in Southeast Asia, using RAPD markers also showed low genetic diversity [15, 16, 40–43].

Simulation for the minimum number of founders estimated that 3–4 individuals settled in the southern and northeastern Thailand represent the founders of the cluster I and II, respectively. *B. flabellifer* is dioecious, and both male and female seeds are required for successful propagation and reproduction. Because there was no mean for sex determination and it takes at least 12 years to verify the sex of individual plants, a number of seeds had to be simultaneously introduced. This number could have been as low as 3–4 seeds to cover a potential outcome for both sexes. This might suggest that, in such case, the introduction was likely occurred through a human activity for exploiting *B. flabellifer*. In addition, this may suggest that, at that period, the human might have learned that *B. flabellifer* requires both male and female trees for fruit production and breeding.

*B. flabellifer* is an invasive plant successfully spreading throughout Thailand and Southeast Asia countries in spite of low genetic diversity. The low genetic diversity of invasive species is commonly referred as the genetic paradox of invasive organisms [44]. The genetic paradox of the *B. flabellifer* may be explained as this plant is useful for human uses, consumption and, perhaps, has tolerant capacities to insects, diseases and environmental challenges at the invading areas. Hence, the successful invasion of *B. flabellifer* was assisted through human activities.

## Conclusions

In this work, we analyzed the genetic data of 230 *B. flabellifer* individuals collected throughout Thailand using 17 EST-SSR and 12 gSSR polymorphic markers. The population was divided into two clusters according to STRUCTURE analysis (delta K, best K = 2) based on three sampling regions, supporting by AMOVA (maximum *F*_CT_ = 0.06266). The cluster I was found predominately in the central and southern Thailand, while the cluster II was found mostly in the northeastern. The minimum number of founders was estimated using the microsatellite data, and it was likely that up to four individuals of each cluster were introduced into two different regions in Thailand. This study proposes the origin of *B. flabellifer* that was introduced into two different locations in the past, and this may also explain the low genetic diversity of the population in Thailand.

## Additional files

Additional file 1: Locations of the *B. flabellifer* samples in Thailand (DOCX 14 kb)

Additional file 2: Representative polyacrylamide electrophoresis gels for the polymorphic loci (DOCX 9268 kb)

Additional file 3: An R script for simulating different numbers of founder genotypes (DOCX 24 kb)

## Abbreviations

AMOVA: Analysis of molecular variance
CTAB: Cetyltrimethylammonium bromide
EST-SSR: Expressed sequence tag- simple sequence repeat
gSSR: Genomic simple sequence repeats
HWE: Hardy–Weinberg equilibrium
LD: Linkage disequilibrium
PIC: Polymorphism Information Content
RAPD: Random amplified polymorphic DNA
