# Supplementary material for: Genetic evidence of multiple invasions and a small number of founders of Asian Palmyra palm (Borassus flabellifer) in Thailand
Source: BMC Genet. 2017 Oct 12;18:88. doi: 10.1186/s12863-017-0554-y (PMC5639744; doi:10.1186/s12863-017-0554-y)
Supplement: Supplementary file 1 — Locations of the B. flabellifer samples in Thailand (DOCX 14 kb) [file 12863_2017_554_MOESM1_ESM.docx]

| **Part of Thailand** | **Province** | **Special code** | **Sample size** | **Location** |
| --- | --- | --- | --- | --- |
| Southern | Songkhla | SK | 18 | 7°17'40.3"N 100°27'39.4"E |
|  | Surat Thani | ST | 13 | 9°15'06.2"N 99°10'30.8"E |
| Central | Phetchaburi | PH | 18 | 13°01'21.8"N 99°54'47.2"E |
|  | Chachoengsao | CH | 5 | 13°44'42.8"N 101°11'48.0"E |
|  | Bangkok | BK | 4 | 13°51'15.0"N 100°49'36.6"E |
|  | Nakhon Pathom | NP | 14 | 13°52'06.3"N 100°10'04.5"E |
|  | Pathum Thani | PT | 6 | 14°01'47.3"N 100°33'00.6"E |
|  | Kanchanaburi | KA | 19 | 14°03'02.7"N 99°37'50.0"E |
|  | Phachinburi | PC | 18 | 14°06'14.8"N 101°31'44.2"E |
|  | Suphan Buri | SB | 6 | 14°31'09.7"N 100°05'16.4"E |
|  | Ang Thong | AT | 5 | 14°39'08.2"N 100°25'56.0"E |
|  | Sing Buri | SI | 7 | 14°48'01.8"N 100°20'12.5"E |
|  | Chainat | CN | 14 | 15°05'57.1"N 100°12'05.6"E |
|  | Nakhon Sawan | NS | 14 | 15°51'33.6"N 100°15'14.4"E |
|  | Sukhothai | SU | 1 | 16 ^o^54'09.6''N 99 ^o^45'37.8''E |
|  | Phitsanulok | PL | 8 | 16 ^o^59'45.3''N 100^o^17'50.6''E |
| North-Eastern | Burirum | BU | 11 | 14°37'04.0"N 102°47'39.0"E |
|  | Sisaket | SS | 9 | 14°58'25.3"N 104°12'33.2"E |
|  | Nakhon-Ratchasima | NR | 10 | 15°13'06.7"N 102°05'03.4"E |
|  | Ubon Ratchathani | UR | 10 | 15°18'30.8"N 105°08'14.1"E |
|  | Amnat Charoen | AC | 5 | 15°37'10.5"N 104°34'01.1"E |
|  | KhonKaen | KK | 3 | 15°47'15.7"N 102°36'25.2"E |
|  | Roi-Et | RE | 6 | 16°05'35.9"N 103°53'37.6"E |
|  | Kalasin | KB | 6 | 16°20'10.3"N 103°38'09.7"E |

**Additional file 1.** Locations of the *B. flabellifer* samples in Thailand.
