# Supplementary material for: Genetic evidence of multiple invasions and a small number of founders of Asian Palmyra palm (Borassus flabellifer) in Thailand
Source: BMC Genet. 2017 Oct 12;18:88. doi: 10.1186/s12863-017-0554-y (PMC5639744; doi:10.1186/s12863-017-0554-y)

**Additional file 2.** Representative polyacrylamide electrophoresis gels for the polymorphic loci.


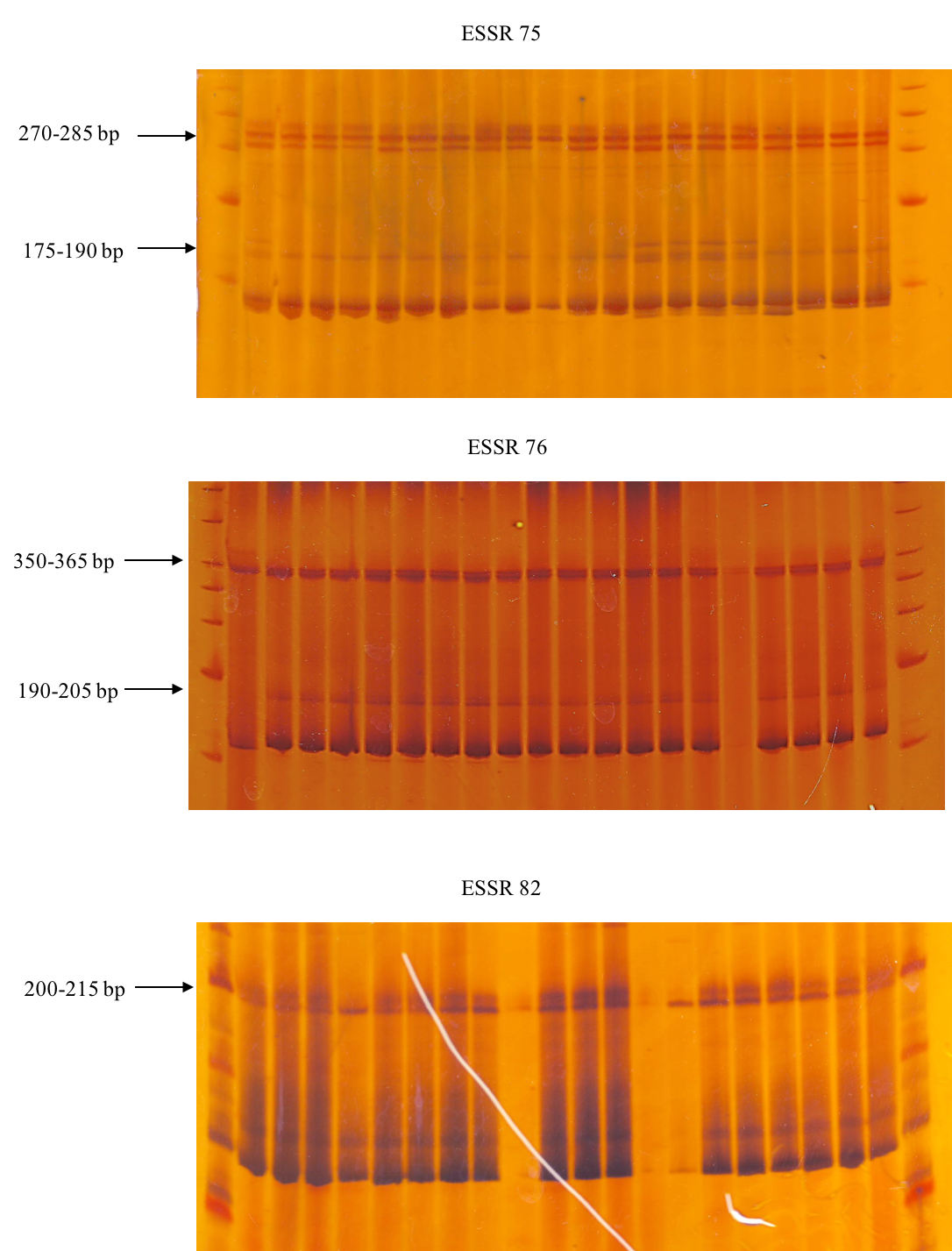


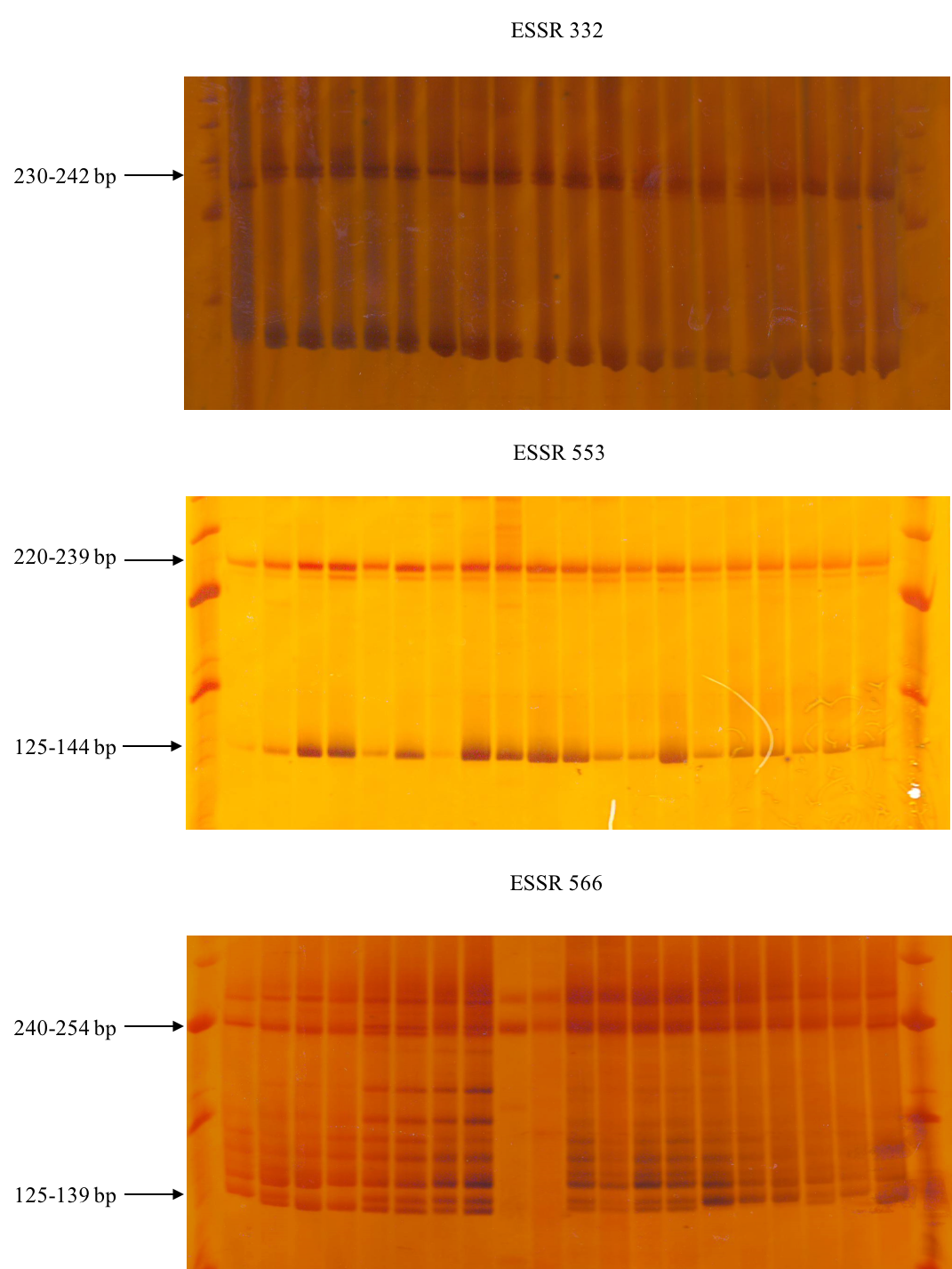


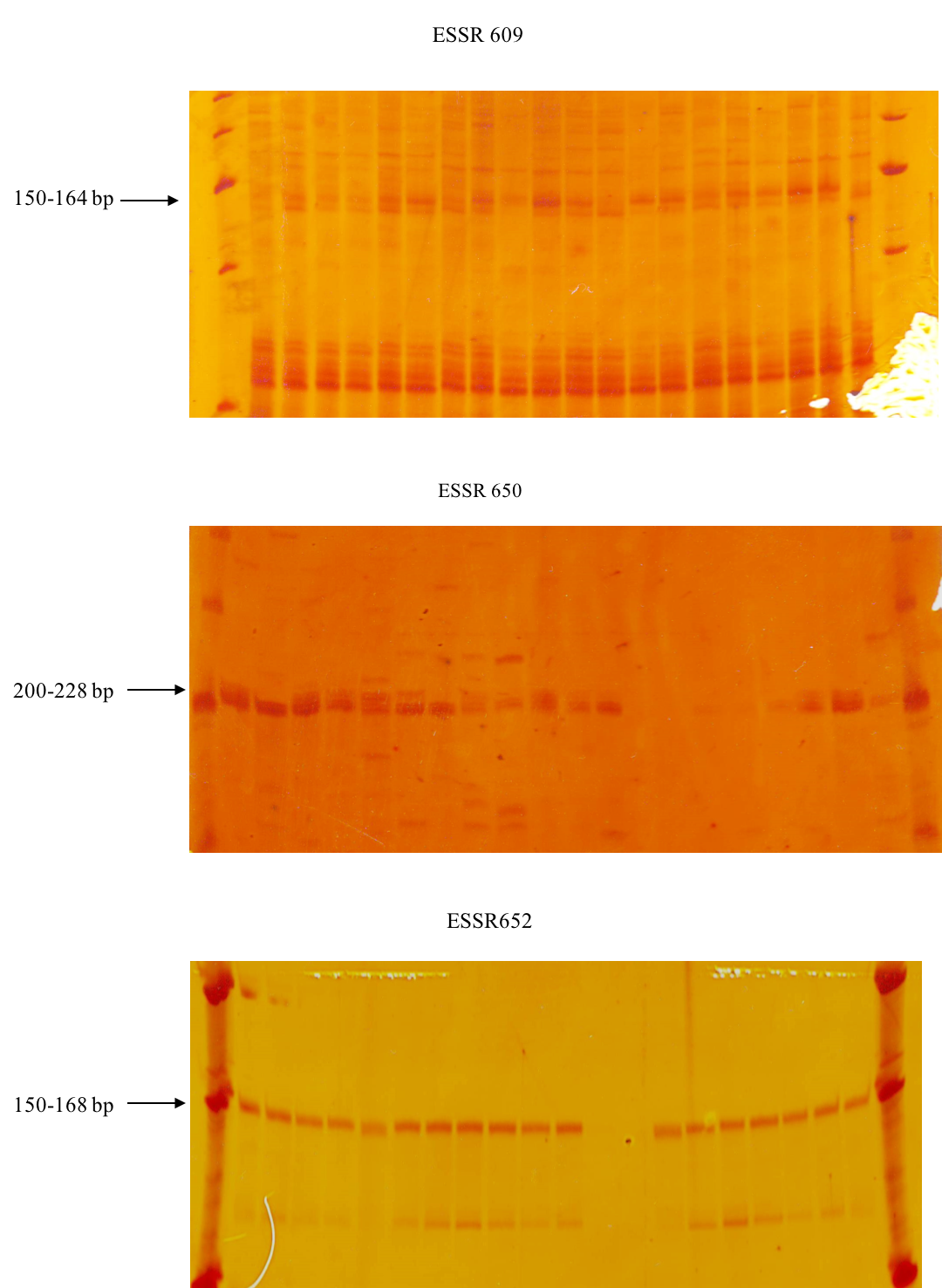


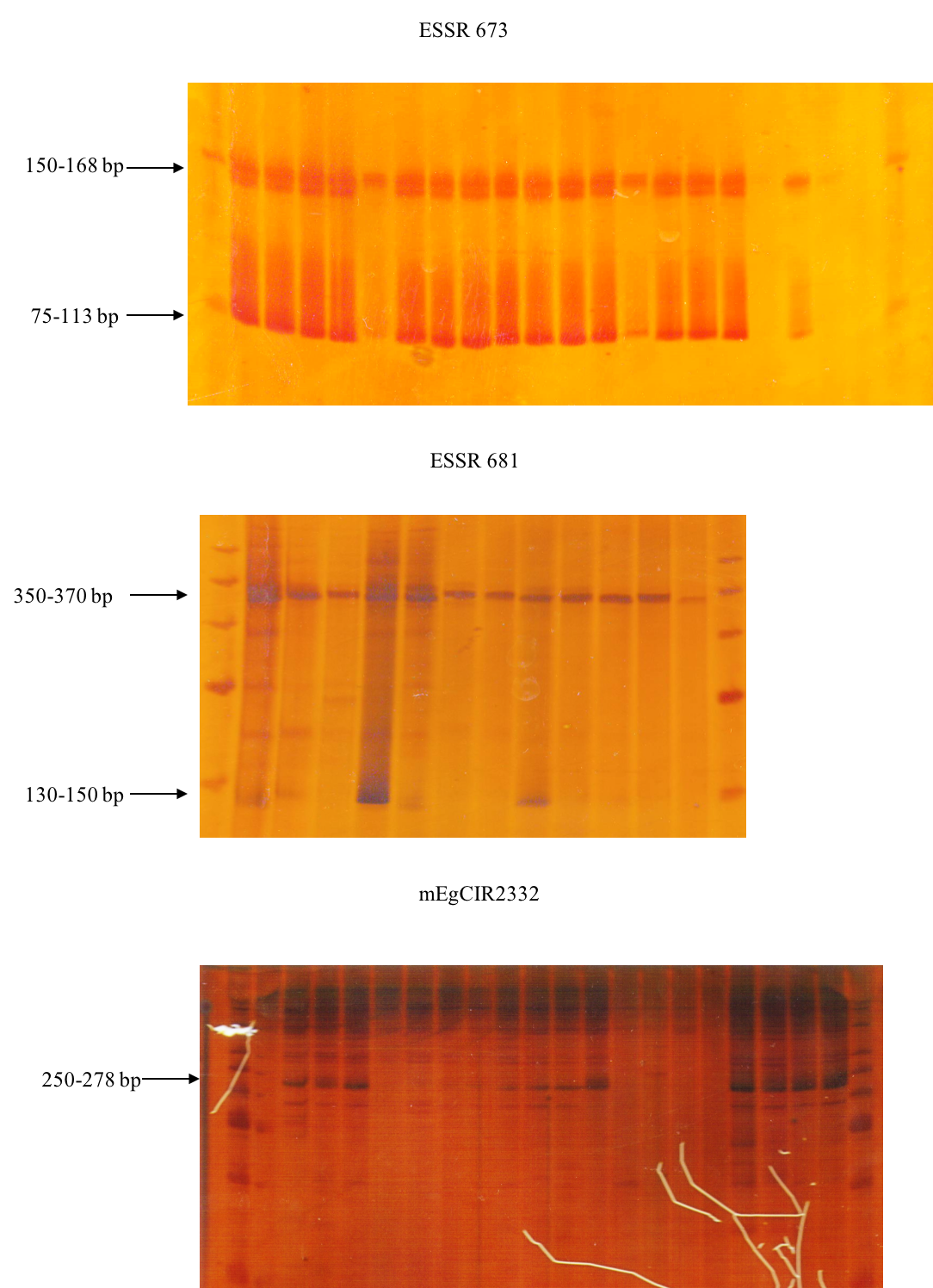


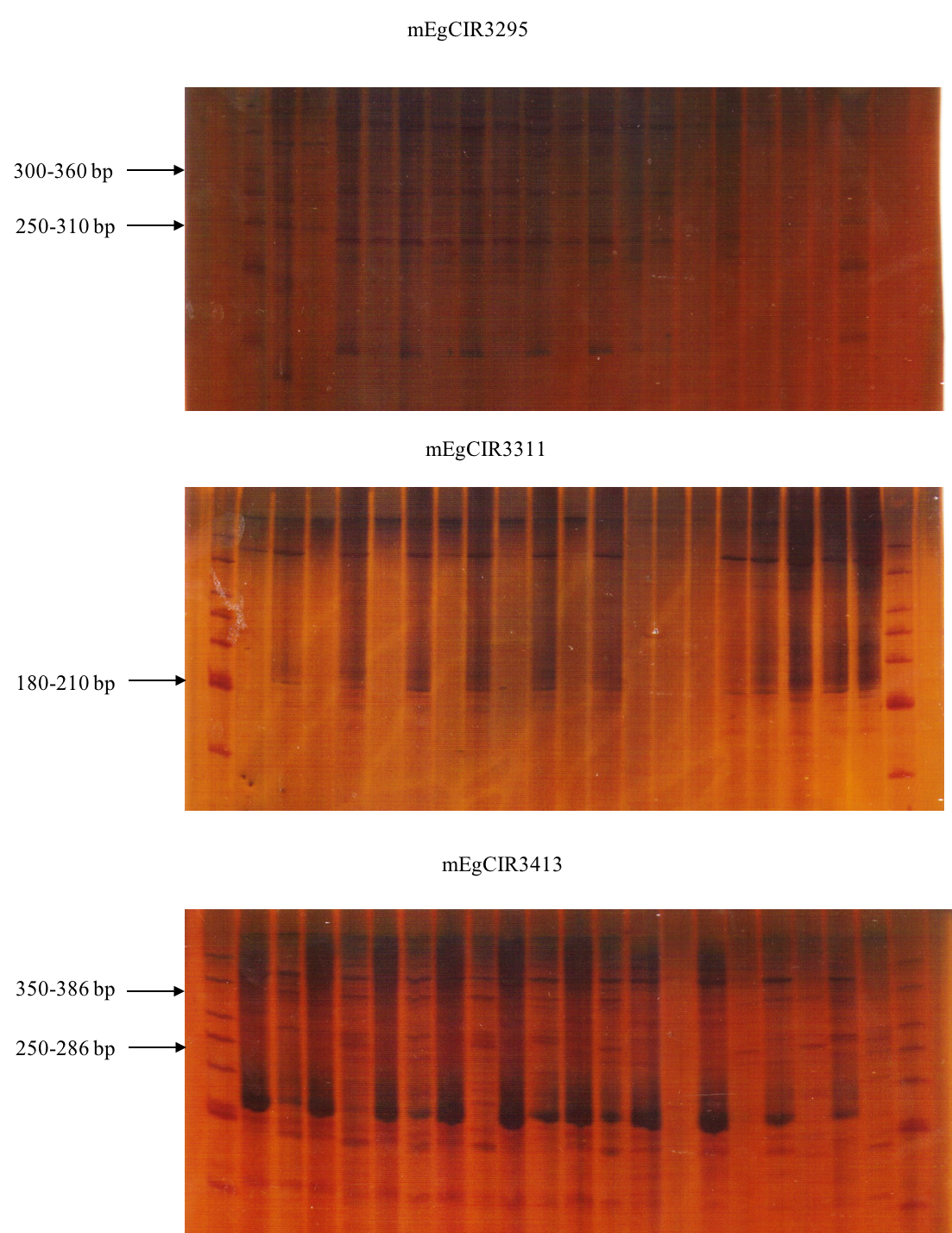


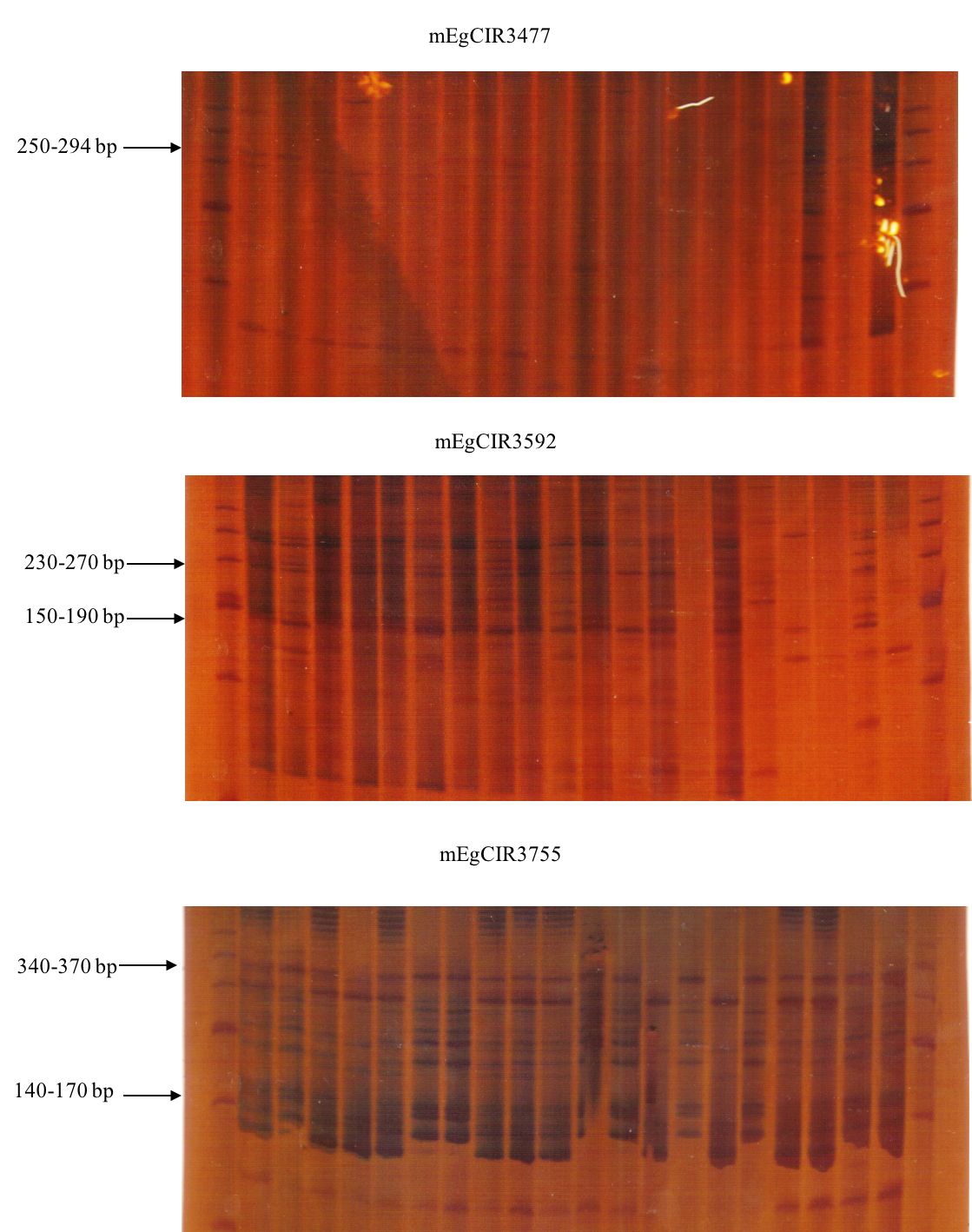


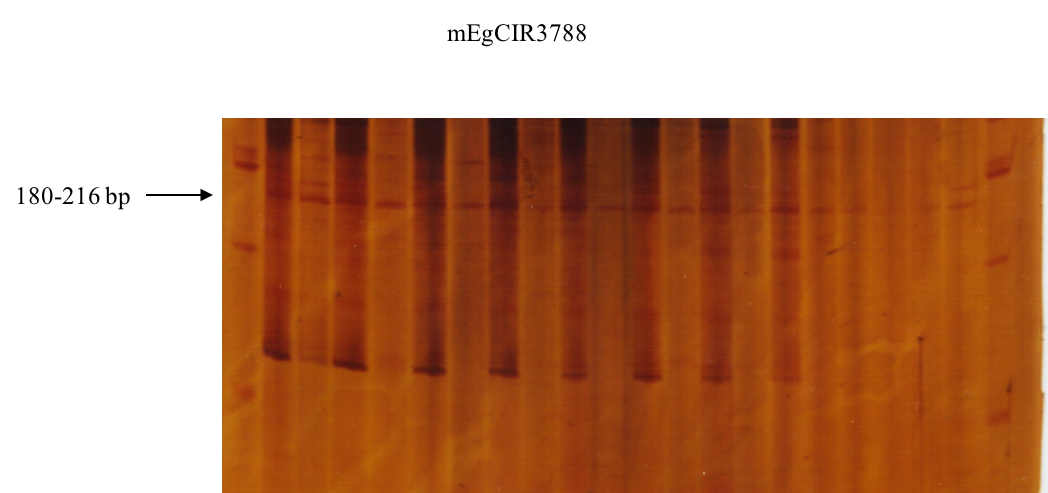

Supplement: Supplementary file 2 — Representative polyacrylamide electrophoresis gels for the polymorphic loci (DOCX 9268 kb) [file 12863_2017_554_MOESM2_ESM.docx]
