# Supplementary material for: Genetic evidence of multiple invasions and a small number of founders of Asian Palmyra palm (Borassus flabellifer) in Thailand
Source: BMC Genet. 2017 Oct 12;18:88. doi: 10.1186/s12863-017-0554-y (PMC5639744; doi:10.1186/s12863-017-0554-y)
Supplement: Supplementary file 3 — An R script for simulating different numbers of founder genotypes (DOCX 24 kb) [file 12863_2017_554_MOESM3_ESM.docx]

**Additional file 3.** An R script for simulating different numbers of founder genotypes.

minFounder = function(dir,firstrow=2,n.its=1000){

f=firstrow-1

print("Please enter name of each population")

pops=scan(,what = "")

print("Please enter sample size of each population")

size=as.integer(scan(,what = ""))

print("Choose program: Original/Remove freq < 0.02/Both (o/r/b)")

choice=scan(,what = "")

result=data.frame(matrix(0,length(pops),4))

colnames(result) <- c("pop","random","allele freq.","equal freq.")

result[,1]=pops

result2=result

datasim<-read.table(dir, header=T, sep="\t")

no.samples=dim(datasim)[1]

noloci=(dim(datasim)[2]-1)/2

locus_positions=seq(1,(noloci*2),2)

population=NULL

lo=is.na(datasim)

for(c in 2:(noloci*2+1)){

for (r in 1:no.samples) {

if(lo[r,c]==T){datasim[r,c]=0}

}}

for(c in 2:(noloci*2+1)){

for (r in 1:no.samples) {

if(datasim[r,c]==0){datasim[r,c]=NA}

}}

random = function(o,allalleles){

collect<-data.frame(matrix(0,n.its,1))

oc=as.data.frame(matrix(0,dim(population[1])*2,noloci))

loc_5=data.frame(matrix(0,1,noloci))

t=1

d=1

prop_samp=0

for(i in 1:n.its){

print(i)

for(l in 1:noloci){

oc[,l]<-sample(o[,l])

}

while(prop_samp!=1){

for(y in 1:noloci){

loc_5[y]<-length(sort(unique(oc[,y][1:(d*2)])))

}

no.alleles_5<-sum(loc_5)

prop_samp<-no.alleles_5/allalleles

d=d+1

}

collect[i,]=d-1

d=1

prop_samp=0

}

return(min(collect))

}

allele = function(OUT,yOUT,allalleles){

collect_2<-data.frame(matrix(0,n.its,1))

tempX=as.data.frame(matrix(1,1,noloci*2))

names(tempX)=names(population)

loc_5=data.frame(matrix(0,1,noloci))

t=1

d=1

prop_samp=0

while(prop_samp!=1){

print(d)

for(i in 1:n.its){

if(prop_samp!=1){

for(W in 1:noloci){

tempX[1:d,(W+(W-1))]=as.character(sample(OUT[which(OUT$Number==(W+(W-1))),3],d,repl=TRUE,prob=yOUT[which(yOUT$Number==(W+(W-1))),5]))

tempX[1:d,(W+(W-1)+1)]=as.character(sample(OUT[which(OUT$Number==(W+(W-1))),3],d,repl=TRUE,prob=yOUT[which(yOUT$Number==(W+(W-1))),5]))

}

for(y in 1:noloci){

loc_5[y]<-length(sort(unique(c(unique(tempX[,t][1:d]),unique(tempX[,t+1][1:d])))))

t=t+2

}

t=1

no.alleles_5<-sum(loc_5)

prop_samp<-no.alleles_5/allalleles

collect_2[i,d]<-prop_samp

}

}

d=d+1

}

names(collect_2)<-1:(d-1)

return(d-1)

}

equal = function(OUT,yOUT,allalleles){

collect_3<-data.frame(matrix(0,n.its,1))

tempX=as.data.frame(matrix(1,1,noloci*2))

names(tempX)=names(population)

loc_5=data.frame(matrix(0,1,noloci))

t=1

d=1

prop_samp=0

while(prop_samp!=1){

print(d)

for(i in 1:n.its){

if(prop_samp!=1){

for(W in 1:noloci){

tempX[1:d,(W+(W-1))]=as.character(sample(OUT[which(OUT$Number==(W+(W-1))),3],d,repl=TRUE,prob=yOUT[which(yOUT$Number==(W+(W-1))),6]))

tempX[1:d,(W+(W-1)+1)]=as.character(sample(OUT[which(OUT$Number==(W+(W-1))),3],d,repl=TRUE,prob=yOUT[which(yOUT$Number==(W+(W-1))),6]))

}

for(y in 1:noloci){

loc_5[y]<-length(sort(unique(c(unique(tempX[,t][1:d]),unique(tempX[,t+1][1:d])))))

t=t+2

}

t=1

no.alleles_5<-sum(loc_5)

prop_samp<-no.alleles_5/allalleles

collect_3[i,d]<-prop_samp

}

}

d=d+1

}

names(collect_3)<-1:(d-1)

return(d-1)

}

for(n in 1:length(pops)){

print(paste("Working on pop",n,"of",length(pops)))

population = datasim[f:(f+size[n]-1),-c(1)]

lnames=colnames(population)

OUT=NULL

o=as.data.frame(matrix(1,dim(population[1])*2,noloci))

names(o)=names(population[(1:noloci)+((1:noloci)-1)])

o[1:(dim(population)[1]),1:noloci]=population[1:(dim(population)[1]),(1:noloci)+((1:noloci)-1)]

o[((dim(population)[1])+1):(dim(o)[1]),1:noloci]=population[1:(dim(population)[1]),(1:noloci)*2]

for (x in locus_positions) {

alleles=c(population[,x],population[,x+1])

alleles2=as.data.frame(table(alleles))

alleles3=cbind(alleles2,alleles2[,2]/sum(alleles2[,2]))

output=cbind(x,lnames[x],alleles3)

OUT=rbind(OUT,output)

}

colnames(OUT) <- c("Number","Locus","allele","count","frequency")

allalleles=dim(OUT)[1]

yOUT=cbind(OUT,OUT[,5])

dimS=data.frame(matrix(0,1,noloci))

for(WW in 1:noloci){

dimS[WW]=length(which(yOUT$Number==(WW+(WW-1))))

yOUT[which(yOUT$Number==(WW+(WW-1))),6]=1/dimS[WW]

}

population2=population

OUT2=OUT[which(OUT$frequency<0.02),]

noloci2=dim(OUT2)[1]

for (a in 1:noloci2) {

population2[which(population2[,OUT2$Number[a]]==OUT2$allele[a]),OUT2$Number[a]]=NA

population2[which(population2[,OUT2$Number[a]+1]==OUT2$allele[a]),OUT2$Number[a]+1]=NA

}

OUT3=NULL

o2=as.data.frame(matrix(1,dim(population2[1])*2,noloci))

names(o2)=names(population2[(1:noloci)+((1:noloci)-1)])

o2[1:(dim(population2)[1]),1:noloci]=population2[1:(dim(population2)[1]),(1:noloci)+((1:noloci)-1)]

o2[((dim(population2)[1])+1):(dim(o2)[1]),1:noloci]=population2[1:(dim(population2)[1]),(1:noloci)*2]

for (x in locus_positions) {

alleles=c(population2[,x],population2[,x+1])

alleles2=as.data.frame(table(alleles))

alleles3=cbind(alleles2,alleles2[,2]/sum(alleles2[,2]))

output=cbind(x,lnames[x],alleles3)

OUT3=rbind(OUT3,output)

}

colnames(OUT3) <- c("Number","Locus","allele","count","frequency")

allalleles2=dim(OUT3)[1]

yOUT2=cbind(OUT3,OUT3[,5])

dimS=data.frame(matrix(0,1,noloci))

for(WW in 1:noloci){

dimS[WW]=length(which(yOUT2$Number==(WW+(WW-1))))

yOUT2[which(yOUT2$Number==(WW+(WW-1))),6]=1/dimS[WW]

}

if(choice=="o"){

print("Result from original data")

result[n,2]=random(o,allalleles)

print(result)

result[n,3]=allele(OUT,yOUT,allalleles)

print(result)

result[n,4]=equal(OUT,yOUT,allalleles)

print(result)

}

if(choice=="r"){

print("Remove freq < 0.02")

result2[n,2]=random(o2,allalleles2)

print(result2)

result2[n,3]=allele(OUT3,yOUT2,allalleles2)

print(result2)

result2[n,4]=equal(OUT3,yOUT2,allalleles2)

print(result2)

}

if(choice=="b"){

print("Result from original data")

result[n,2]=random(o,allalleles)

print(result)

result[n,3]=allele(OUT,yOUT,allalleles)

print(result)

result[n,4]=equal(OUT,yOUT,allalleles)

print(result)

print("Remove freq < 0.02")

result2[n,2]=random(o2,allalleles2)

print(result2)

result2[n,3]=allele(OUT3,yOUT2,allalleles2)

print(result2)

result2[n,4]=equal(OUT3,yOUT2,allalleles2)

print(result2)

}

f=f+size[n]

}

if(choice=="o"){

write.table(result,"result.txt",sep="\t",row.names = F)

}

if(choice=="r"){

write.table(result2,"result-0.02.txt",sep="\t",row.names = F)

}

if(choice=="b"){

write.table(result,"result.txt",sep="\t",row.names = F)

write.table(result2,"result-0.02.txt",sep="\t",row.names = F)

}

}
